# Case-Finding Strategies for Drug-Resistant Tuberculosis: Protocol for a Scoping Review

**DOI:** 10.2196/40009

**Published:** 2022-12-15

**Authors:** Susanna S Van Wyk, Marriott Nliwasa, James A Seddon, Graeme Hoddinott, Lario Viljoen, Emmanuel Nepolo, Gunar Günther, Nunurai Ruswa, Hsien-Ho Lin, Stefan Niemann, Neel R Gandhi, N Sarita Shah, Mareli Claassens

**Affiliations:** 1 Centre for Evidence Based Health Care, Division of Epidemiology and Biostatistics, Department of Global Health Stellenbosch University Cape Town South Africa; 2 Helse Nord Tuberculosis Initiative, Kamuzu University of Health Sciences Blantyre Malawi; 3 Desmond Tutu Tuberculosis Centre, Department of Paediatrics and Child Health Faculty of Medicine and Health Sciences Stellenbosch University Cape Town South Africa; 4 Department of Infectious Diseases Imperial College London London United Kingdom; 5 Department of Human, Biological and Translational Medical Science School of Medicine University of Namibia Windhoek Namibia; 6 Department of Pulmonary Medicine, Inselspital, Bern University Hospital University of Bern Bern Switzerland; 7 Ministry of Health and Social Services Windhoek Namibia; 8 Institute of Epidemiology and Preventive Medicine National Taiwan University Taipei Taiwan; 9 Molecular and Experimental Mycobacteriology Group Forschungszentrum Borstel Borstel Germany; 10 National Reference Center for Mycobacteria Forschungszentrum Borstel Borstel Germany; 11 German Centre for Infection Research (DZIF) Partner Site Hamburg-Lübeck-Borstel-Riems Borstel Germany; 12 Department of Epidemiology Rollins School of Public Health Emory University Atlanta, GA United States; 13 Department of Global Health Rollins School of Public Health Emory University Atlanta, GA United States

**Keywords:** drug-resistant tuberculosis, case finding, public health, drug, drug-resistant, tuberculosis, treatment, clinical, transmission, acceptability, feasibility, research, policy, literature, model, data, systematic review, case-finding strategies

## Abstract

**Background:**

Transmission of drug-resistant tuberculosis (DR-TB) is ongoing. Finding individuals with DR-TB and initiating treatment as early as possible is important to improve patient clinical outcomes and to break the chain of transmission to control the pandemic. To our knowledge systematic reviews assessing effectiveness, cost-effectiveness, acceptability, and feasibility of different case-finding strategies for DR-TB to inform research, policy, and practice have not been conducted, and it is unknown whether enough research exists to conduct such reviews. It is unknown whether case-finding strategies are similar for DR-TB and drug-susceptible TB and whether we can draw on findings from drug-susceptible reviews to inform decisions on case-finding strategies for DR-TB.

**Objective:**

This protocol aims to describe the available literature on case-finding for DR-TB and to describe case-finding strategies.

**Methods:**

We will screen systematic reviews, trials, qualitative studies, diagnostic test accuracy studies, and other primary research that specifically sought to improve DR-TB case detection. We will exclude studies that invited individuals seeking care for TB symptoms, those including individuals already diagnosed with TB, or laboratory-based studies. We will search the academic databases including MEDLINE, Embase, The Cochrane Library, Africa-Wide Information, CINAHL, Epistemonikos, and PROSPERO with no language or date restrictions. We will screen titles, abstracts, and full-text articles in duplicate. Data extraction and analyses will be performed using Excel (Microsoft Corp).

**Results:**

We will provide a narrative report with supporting figures or tables to summarize the data. A systems-based logic model, developed from a synthesis of case-finding strategies for drug-susceptible TB, will be used as a framework to describe different strategies, resulting pathways, and enhancements of pathways. The search will be conducted at the end of 2021. Title and abstract screening, full text screening, and data extraction will be undertaken from January to June 2022. Thereafter, analysis will be conducted, and results compiled.

**Conclusions:**

This scoping review will chart existing literature on case-finding for DR-TB—this will help determine whether primary studies on effectiveness, cost-effectiveness, acceptability, and feasibility of different case-finding strategies for DR-TB exist and will help formulate potential questions for a systematic review. We will also describe case-finding strategies for DR-TB and how they fit into a model of case-finding pathways for drug-susceptible TB. This review has some limitations. One limitation is the diverse, inconsistent use of intervention terminology within the literature, which may result in missing relevant studies. Poor reporting of intervention strategies may also cause misunderstanding and misclassification of interventions. Lastly, case-finding strategies for DR-TB may not fit into a model developed from strategies for drug-susceptible TB. Nevertheless, such a situation will provide an opportunity to refine the model for future research. The review will guide further research to inform decisions on case-finding policies and practices for DR-TB.

**International Registered Report Identifier (IRRID):**

DERR1-10.2196/40009

## Introduction

With the emergence of *Mycobacterium tuberculosis* strains resistant to first-line antituberculosis drugs, strategies to control tuberculosis (TB) became even more challenging [1]. It is estimated that almost half a million people have developed rifampicin-resistant TB, of whom 78% had multidrug-resistant TB in 2019 [2]. Although drug-resistant TB (DR-TB) is not as prevalent as drug-susceptible TB, it is more difficult to diagnose, treatment is longer and more toxic, outcomes are worse, and costs are higher. Overall, 67%-100% of people with DR-TB in their households face catastrophic costs (total costs equivalent to >20% of their annual household income) [2].

Finding individuals with DR-TB and initiating treatment as early as possible is important to improve patient clinical outcomes and to break the chain of transmission to help control the pandemic. But despite new diagnostic technologies, only 38% of the estimated number of people who developed DR-TB initiated treatment in 2019 [2,3].

TB can be detected after an individual presents passively to health services or through one of several different screening pathways depending on the case-finding strategy of a TB program [4]. Pathways can also be enhanced via several activities such as health promotion in the community, improved access to TB diagnostic services or training of health workers to identify presumptive TB at general health services. Multiple activities often result in complex interventions and heterogeneous trials, which are difficult to meta-analyze in systematic reviews [5,6].

To our knowledge, systematic reviews assessing effectiveness, cost-effectiveness, acceptability, and feasibility of different case-finding strategies for DR-TB to inform research, policy, and practice have not been conducted, and it is unknown whether enough research exists to conduct such reviews. It is also unknown whether case-finding strategies are similar for DR-TB and drug-susceptible TB and whether we can draw on findings from reviews on drug-susceptible TB to inform decisions on case-finding strategies for DR-TB.

We therefore aim to conduct a scoping review to chart existing literature on case finding for DR-TB and identify priority questions for a systematic review [7,8]. We will also describe existing strategies and how they fit into a model of case-finding pathways for drug-susceptible TB.

## Methods

The Arksey and O’Malley framework [9,10], the Joanna Briggs Institute scoping review methodology [8], and the Preferred Reporting Items for Systematic reviews and Meta-Analyses extension for Scoping Reviews [11] will guide the methods for this scoping review.

### Defining of the Research Question

The question for our review is as follows: what literature is available on case-finding for DR-TB and which case-finding strategies are described? We will screen studies that have sought to improve case detection for DR-TB.

### Identification of Relevant Studies

Table 1 outlines the eligibility criteria for this scoping review.

**Table 1:** Eligibility criteria.

Domain	Included	Excluded
Participants	Participate regardless of symptoms; for example, contacts, people living with HIV attending HIV care, and whole communities	Individuals with tuberculosis (TB) symptoms seeking care, individuals diagnosed with TB, and laboratory samples and isolates
Concept or intervention	Strategies specifically aiming to improve or enhance participants’ pathways to drug-resistant TB case detection	Intervention strategies aiming to improve finding of TB cases in general, even if they report the yield of drug-resistant TB cases
Outcome	Reporting the yield of drug-resistant TB	Do not report the yield of drug-resistant TB
Context	Community and primary, secondary, or tertiary care centers	Laboratory-based
Study design	Primary studies; systematic reviews; qualitative studies, where the experiences of individuals who receive the intervention or those who provide the intervention are investigated; studies of diagnostic test accuracy if they describe a drug-resistant TB screening strategy; and trials comparing different screening or diagnostic tools within a case-finding intervention for drug-resistant TB	Meta-reviews (review of reviews); narrative reviews; editorials; opinion articles; meeting summaries; guidelines; prevalence surveys, except if the survey includes an intervention strategy to specifically find drug-resistant TB cases; and conference abstracts

With assistance from an information specialist, we will search MEDLINE (PubMed), Embase (Ovid), and The Cochrane Library because they are top academic databases for biomedical research, medicine, and health care; Africa-Wide Information (EBSCOhost) owing to the high prevalence of TB in sub-Saharan Africa; CINAHL (EBSCOhost) because nurses and allied health staff are often the ones carrying out the actual case-finding; and Epistemonikos and PROSPERO to complement the search for systematic reviews. To obtain the highest possible yield, we will use no language or date restrictions; however, if we are not able to translate the articles, we will report them under excluded articles with reasons for exclusion. Reference lists of included studies will be searched.

The preliminary search string will include combinations of the following 3 domains: terms related to “tuberculosis,” terms related to “drug resistance,” and terms related to “case finding,” “case detection,” “screening,” “contact investigation,” and “contact tracing.”

Appropriate MeSH (Medical Subject Headings) terms will be added to the different databases. Search strategies from each electronic database are detailed in Multimedia Appendix 1. The search strategy will be piloted and refined in consultation with an information specialist. We will also contact experts working in the field to collect information about ongoing primary research or relevant research missed by the electronic search.

### Study Selection

We will use Rayyan systematic review software [12] to screen titles, abstracts, and full-text articles. We will use the “blind” function in Rayyan for screening, except when resolving conflicts. Four reviewers (SvW, MN, LV, and MC) will screen abstracts in duplicate for inclusion. They will resolve conflicts via discussion and meet at the beginning of and after screening 50 abstracts to discuss challenges and possible refinement of the search strategy. Three reviewers (SvW, LV, and MN) will then screen full-text articles for inclusion. Disagreements will be resolved with a third reviewer (MC) to determine the final studies for inclusion.

### Charting of the Data

We will develop a data extraction form in Excel (Microsoft Corp). The data extraction form will be applied to all primary research reports to collect standard information on each study. Information will include the following: authors, journal name, and year of publication; aim or purpose of the research; study design; country characteristics (including income, TB prevalence, HIV prevalence, and urban or rural setting); participants’ characteristics (including age, sex, HIV status, and other reported risk factors); target group and how the group was identified, if applicable; interventions (including all components [ie, activities] of the intervention, types of providers, and screening and diagnostic tools used); treatment support, including preventive therapy; and outcomes assessed.

Five authors (SvW, MN, LV, MC, and GH) will extract the data (1 author per paper). A second reviewer (SvW or MC) will check extracted data from each study. The data extraction team and other coauthors will meet regularly after each round of screening of 5-10 studies to determine whether their approach is consistent and in line with the research question.

### Patient and Public Involvement

This paper describes the protocol for a scoping review; hence, we did not deem it appropriate to involve patients or the public in the design, conduct, reporting, or dissemination plans of our research.

## Results

### Collating, Summarizing, and Reporting of the Results

We will provide a narrative report with supporting figures or tables to summarize the data. Textbox 1 contains the definitions we will use in charting, collating, summarizing, and reporting our results.

**Definitions.**

**Definitions used in the scoping review:**

**Figure 1:**
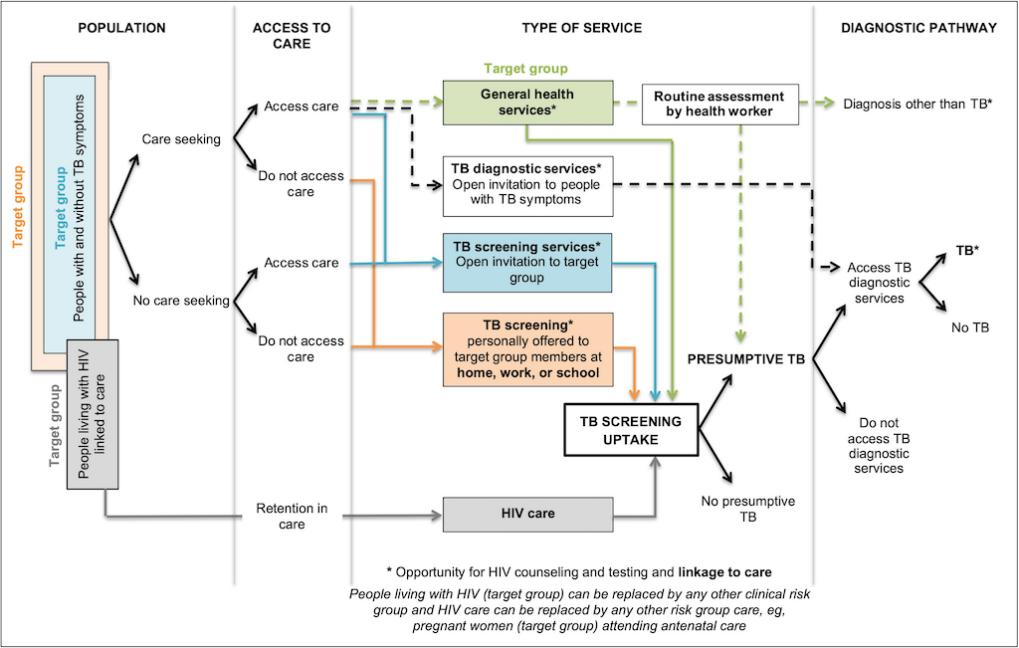
A systems-based logic model depicting types of services and associated pathways to tuberculosis (TB) case detection.

- **Drug-resistant tuberculosis (TB):** all types of drug-resistant TB, including single drug–resistant TB, multidrug-resistant TB, extensively drug-resistant TB, and any other drug-resistant TB reported by the authors.
- **Systematic screening for active TB:** “The systematic identification of people with suspected (presumptive) active TB, in a predetermined target group, using tests, examinations or other procedures that can be applied rapidly. Among those screened positive, the diagnosis needs to be established by one or several diagnostic tests and additional clinical assessments, which together have high accuracy” [3].
- **A screening tool:** tests, examinations, or other procedures used for systematic screening for active TB. Examples of TB screening tools include a structured symptom-based questionnaire, chest radiography, or an algorithm [4]. Algorithms may include sequential or parallel tests. With sequential tests, only those who screen positive with the initial test receive a second test. With parallel tests, those who screen positive on any of the tests are regarded as screen positives.
- **A diagnostic tool:** tests, examinations, or other procedures used to establish a diagnosis of TB in people identified with presumptive TB. Examples of TB diagnostic tools include a clinical algorithm, sputum smear microscopy, the Xpert MTB/RIF test, or cultures [4].
- **TB symptoms:** any TB symptom, including cough, fever, night sweats, weight loss, or a combination of TB symptoms as defined by the study authors.
- **Care seeking:** people seeking care for a perceived health problem.
- **TB care seeking:** people seeking care specifically for TB symptoms.
- **A risk group:** any group of people in whom the prevalence or incidence of TB is significantly higher than that in the general population. Examples of risk groups include a whole population within a geographical area or TB contacts [3].
- **A clinical risk group:** individuals diagnosed with a specific disease or condition that increases their risk for TB; for example, people living with HIV.
- **Presumptive TB:** presumptive TB is identified when a provider identifies a patient with suspected active TB. In the context of screening, a person who screens positive is a presumptive TB case.
- **Passive case finding, passive case finding with an element of systematic screening, triage, enhanced case finding, active case finding, contact tracing or contact investigation, and intensified case finding:** some of these definitions overlap and are used inconsistently within the literature. We will therefore use our logic model (Figure 1) to clearly describe pathways rather than labeling them with these terms.

A systems-based logic model developed from a synthesis of case-finding strategies for drug-susceptible TB (Figure 1) will be used as a framework to describe different strategies and resulting pathways [13]. As an example, a target group—for example, household contacts from a TB source case—can be invited to the clinic if they develop symptoms (care-seeking pathway); they can be invited regardless of symptoms (blue screening pathway); or they can be screened at home, resulting in yet a different pathway (orange pathway). Enhancements to pathways will also be described and may include enhanced care-seeking (eg, health promotion), improved access to care for those seeking care (eg, mobile clinics), improved access to TB screening (eg, incentives), improved identification of presumptive TB by health workers (eg, training of health workers and incentives), and improved access to TB diagnostic services for individuals identified with presumptive TB (eg, transport, sputum collection in the community, and mobile laboratories). For screening pathways, we will report on target groups and screening and diagnostic tools used.

Quality appraisal will not be conducted because this is a scoping review and our interest is in the existing evidence base, regardless of study design and quality.

### Timeline

The search will be conducted at the end of 2021. Title and abstract screening, full text screening, and data extraction will be carried out from January to June 2022. Thereafter, analysis will be conducted and results written up.

## Discussion

### Expected Findings

This scoping review will chart existing literature on case finding for DR-TB; this will help us determine whether primary studies on effectiveness, cost-effectiveness, acceptability, and feasibility of different DR-TB case-finding strategies exist. This will assist us in formulating potential questions for a systematic review. We will also describe case-finding strategies for DR-TB and how they fit into a model of case-finding strategies for drug-susceptible TB.

### Strengths and Limitations

Our multidisciplinary review team consists of researchers with extensive experience in TB-related research and the conduct of systematic reviews and qualitative evidence synthesis. Their experience would be invaluable in collating and summarizing diverse literature in a sensible way. Another strength of our review is the use of a systems-based logic model that was developed from a synthesis of case-finding strategies for drug-susceptible TB. The model will help construct meaningful pathway descriptions for possible comparisons in future research and to assess whether case-finding pathways for DR-TB are similar to those for drug-susceptible TB.

One limitation to the review is the diverse, inconsistent use of intervention terminology within the literature, which may result in missing relevant studies. Although we cannot completely overcome this problem, we will work with an information specialist to pilot and optimize our search strategy, and we will discuss potential refinements to our search at regular meetings during the screening phase. Poor reporting of intervention strategies may also cause misunderstanding and misclassification of interventions. However, we do not assess effectiveness as an outcome; therefore, bias due to misclassification would be a minor issue. Lastly, case-finding strategies for DR-TB may not fit into a model developed from those for drug-susceptible TB. Nevertheless, such a situation will provide an opportunity to refine the model for future research.

### Conclusions

This scoping review will chart the existing body of literature on case-finding strategies for DR-TB and will guide further research to inform decisions regarding case-finding policies and practices for DR-TB.

## Appendix

Multimedia Appendix 1 Search strategies from electronic databases.

## Abbreviations

DR-TB: drug-resistant tuberculosis
MeSH: Medical Subject Headings
TB: tuberculosis

## Data Availability

Data sets will be deposited in a publicly available repository, if applicable.
