# Supplementary material for: Case-Finding Strategies for Drug-Resistant Tuberculosis: Protocol for a Scoping Review
Source: JMIR Res Protoc. 2022 Dec 15;11(12):e40009. doi: 10.2196/40009 (PMC9801265; doi:10.2196/40009)
Supplement: Multimedia Appendix 1 [file resprot_v11i12e40009_app1.doc]

Medline PubMed

#1 "Tuberculosis, Multidrug-Resistant"[Mesh]

#2 "Extensively Drug-Resistant Tuberculosis"[Mesh]

#3 ("tuberculosis"[MeSH] OR "Mycobacterium tuberculosis"[MeSH] OR TB[Title/Abstract] OR tuberculosis[Title/Abstract]) AND (drug resist*[Title/Abstract] OR MDR[Title/Abstract] OR DR[Title/Abstract] OR XDR[Title/Abstract])

#4 DR-TB[Title/Abstract] OR MDR-TB[Title/Abstract] OR XDR-TB[Title/Abstract]

#5 Case* detection[Title/Abstract]

#6 Case* finding[Title/Abstract]

#7 Screening[Title/Abstract]

#8 contact investigation[Title/Abstract] OR contact tracing[Title/Abstract] OR contact finding[Title/Abstract]

#9 #1 OR #2 OR #3 OR #4

#10 #5 OR #6 OR #7 OR #8

#11 #9 AND #10

Embase

Embase 1947-Present, updated daily

1 multidrug resistant tuberculosis/

2 extensively drug resistant tuberculosis/

3 Mycobacterium tuberculosis/ or tuberculosis/

4 (TB or tuberculosis).tw.

5 drug resistance.mp. or drug resistance/

6 drug resistant.tw.

7 3 or 4

8 5 or 6

9 7 and 8

10 (DR-TB or MDR-TB or XDR-TB).tw.

11 1 or 2 or 9 or 10

12 case detection.mp.

13 case finding.mp. or case finding/

14 screening.tw.

15 contact investigation.mp.

16 contact tracing.mp. or contact examination/

17 contact finding.mp.

18 12 or 13 or 14 or 15 or 16 or 17

19 11 and 18

20 (rat or rats or mouse or mice or swine or porcine or murine or sheep or lambs or pigs or piglets or rabbit or rabbits or cat or cats or dog or dogs or cattle or bovine or monkey or monkeys or trout or marmoset*).ti. and animal experiment/

21 Animal experiment/ not (human experiment/ or human/)

22 20 or 21

23 19 not 22

The Cochrane Library

ID Search Hits

#1 MeSH descriptor: [Tuberculosis, Multidrug-Resistant] explode all trees

#2 MeSH descriptor: [Extensively Drug-Resistant Tuberculosis] explode all trees

#3 MeSH descriptor: [Tuberculosis] explode all trees

#4 MeSH descriptor: [Mycobacterium tuberculosis] explode all trees

#5 (TB):ti,ab,kw

#6 (tuberculosis):ti,ab,kw

#7 #3 OR #4 OR #5 OR #6

#8 (drug resist*):ti,ab,kw

#9 (MDR):ti,ab,kw

#10 (DR):ti,ab,kw

#11 (XDR):ti,ab,kw

#12 #8 OR #9 OR #10 OR #11

#13 #7 AND #12

#14 (DR-TB):ti,ab,kw

#15 (MDR-TB):ti,ab,kw

#16 (XDR-TB):ti,ab,kw

#17 #1 OR #2 OR #13 OR #14 OR #15 OR #16

#18 (Case* detection):ti,ab,kw

#19 (Case* finding):ti,ab,kw

#20 (Screening):ti,ab,kw

#21 (contact investigation):ti,ab,kw

#22 (contact tracing):ti,ab,kw

#23 (contact finding):ti,ab,kw

#24 #18 OR #19 OR #20 #21 OR #22 OR #23

#25 #17 AND #24 in Cochrane Reviews, Cochrane Protocols, Trials

Africa-Wide Information (EBSCOhost)

| **#** | **Query** |
| --- | --- |
| S11 | S5 AND S10 |
| S10 | S6 OR S7 OR S8 OR S9 |
| S9 | TI ( contact investigation OR contact tracing OR contact finding ) OR AB ( contact investigation OR contact tracing OR contact finding ) |
| S8 | TI screening OR AB screening |
| S7 | TI ( case finding or cases finding ) OR AB ( case finding or cases finding ) |
| S6 | TI ( case detection or cases detection ) OR AB ( case detection or cases detection ) |
| S5 | S3 OR S4 |
| S4 | TI ( DR-TB OR MDR-TB OR XDR-TB ) OR AB ( DR-TB OR MDR-TB OR XDR-TB ) |
| S3 | S1 AND S2 |
| S2 | TI ( drug resistant OR drug resistance ) OR AB ( drug resistant OR drug resistance ) |
| S1 | TI ( tuberculosis OR TB ) OR AB ( tuberculosis OR TB ) |

CINAHL (EBSCOhost)

| **#** | **Query** |
| --- | --- |
| S22 | S12 AND S21 |
| S21 | S13 OR S14 OR S15 OR S16 OR S17 OR S18 OR S19 OR S20 |
| S20 | TI contact investigation OR contact tracing OR contact finding |
| S19 | AB contact investigation OR contact tracing OR contact finding |
| S18 | AB screening |
| S17 | TI screening |
| S16 | TI case finding or cases finding |
| S15 | AB case finding or cases finding |
| S14 | AB case detection or cases detection |
| S13 | TI case detection or cases detection |
| S12 | S1 OR S2 OR S8 OR S9 OR S11 |
| S11 | S5 AND S10 |
| S10 | S6 OR S7 |
| S9 | TI DR-TB OR MDR-TB OR XDR-TB |
| S8 | AB DR-TB OR MDR-TB OR XDR-TB |
| S7 | AB drug resistant OR drug resistance |
| S6 | TI drug resistant OR drug resistance |
| S5 | S3 OR S4 |
| S4 | AB tuberculosis OR tb |
| S3 | TI tuberculosis OR tb |
| S2 | MJ tuberculosis drug resistant |
| S1 | MJ tuberculosis multidrug resistant |

Epistemonikos

(title:((title:((tuberculosis OR TB) AND (drug resistant OR drug resistance) AND (case detection OR case finding OR screening OR contact investigation OR contact tracing OR contact finding)) OR abstract:((tuberculosis OR TB) AND (drug resistant OR drug resistance) AND (case detection OR case finding OR screening OR contact investigation OR contact tracing OR contact finding)))) OR abstract:((title:((tuberculosis OR TB) AND (drug resistant OR drug resistance) AND (case detection OR case finding OR screening OR contact investigation OR contact tracing OR contact finding)) OR abstract:((tuberculosis OR TB) AND (drug resistant OR drug resistance) AND (case detection OR case finding OR screening OR contact investigation OR contact tracing OR contact finding)))))

PROSPERO

**(tuberculosis OR TB) AND (drug resistant OR drug resistance) AND (case detection OR case finding OR screening OR contact investigation OR contact tracing OR contact finding)**
